# Emicizumab prophylaxis beyond clinical trials: a multicenter, prospective real-world study of pediatric hemophilia patients with and without inhibitors

**DOI:** 10.1007/s00431-026-06872-z

**Published:** 2026-04-11

**Authors:** Laila M. Sherief, Osama Elagamy, Ahmad Darwish, Nada K. Soliman, Mohamed Rashad Elgendy, Amira Nazim, Ahmed Sobhi

**Affiliations:** 1 Pediatric Hematology and Oncology Unit, Pediatric Department, Faculty of Medicine, Zagazig University, Zagazig, Egypt; 2 Department of Pediatrics, Faculty of Medicine, Kafrelsheikh University, Kafrelsheikh, Egypt; 3 Pediatric Hematology and Oncology Unit, Pediatric Department, Faculty of Medicine, Mansoura University, Mansoura, Egypt; 4 Pediatric Department, Mabara Health Insurance Hospital, Zagazig, Egypt; 5 Pediatric Department, Mansoura Health Insurance Hospital, Mansoura, Egypt

**Keywords:** Hemophilia A, Emicizumab, FVIII inhibitors, Joint health outcomes

## Abstract

Emicizumab has improved prophylactic management of hemophilia A (HA), offering effective bleed prevention independent of factor VIII (FVIII) inhibitor status. However, prospective real-world pediatric data from low- and middle-income settings remain limited. We conducted a prospective cohort study of 72 children (< 18 years) with HA receiving subcutaneous emicizumab prophylaxis for 12 months. The cohort comprised 70 patients with severe HA and 2 with moderate HA with severe bleeding phenotype. Patients included both inhibitor-positive and inhibitor-negative children. Outcomes included annualized bleeding rate (ABR), joint health assessed by Hemophilia Joint Health Score version 2.1 (HJHS 2.1), and functional capacity measured by the Functional Independence Score in Hemophilia (FISH). The. median ABR decreased from 40 (IQR 36–48) at baseline to 0 (IQR 0–0) after 12 months (*P* < 0.001), with 79.16% of children experiencing zero treated bleeds. AJBR was 0 in 86.1% of patients, and all target joints resolved by 12 months. The median HJHS improved from 15 (IQR 7–22) to 9 (IQR 3–14) (*P* < 0.001), and mean FISH score increased from 20.65 ± 4.25 to 26.02 ± 3.57 (*P* < 0.001), with greatest improvements in squatting, running, and stair climbing. Reductions in ABR, HJHS, and FISH were comparable between inhibitor-positive and inhibitor-negative patients.

*Conclusion*: Emicizumab prophylaxis provides profound bleed control and meaningful improvements in joint and functional outcomes in children with HA, regardless of inhibitor status, supporting its use as standard pediatric prophylaxis in routine clinical practice.

**Table:** 

**What is Known:** • *Emicizumab effectively reduces bleeding in children with hemophilia A.* • *Prospective real-world pediatric data in low- and middle-income settings remain limited.* **What is New:** • *This study showed near-complete bleed control, full target joint resolution.* • *Comparable benefits were observed in children with and without inhibitors in a prospective real-world setting.*

**Supplementary Information:**

The online version contains supplementary material available at 10.1007/s00431-026-06872-z.

## Introduction

Hemophilia A (HA) is a congenital bleeding disorder caused by deficient or dysfunctional factor VIII (FVIII), leading to inadequate thrombin generation and predisposing to either spontaneous or trauma-related hemorrhage [1]. Historically, the prophylactic basis of HA management relied on regular intravenous infusions of FVIII concentrates to maintain protective trough levels and prevent joint and muscle bleeding [2]. However, this strategy posed substantial clinical and practical challenges. Infusion schedules created a considerable treatment burden for patients and caregivers, while repeated venous access in young children is considered a persistent obstacle [3, 4]. In addition, adherence to prophylactic FVIII infusions frequently declined over time, impacting treatment effectiveness and contributing to progressive joint disease [5]. FVIII alloantibody inhibitors develop in up to 30% of individuals with severe HA, which neutralize infused FVIII and dramatically reduce the efficacy of conventional replacement therapy. This causes long-term bleeding prevention to be considerably more challenging [6, 7].

Emicizumab is considered a safe, effective, and less burdensome prophylaxis option, especially for patients with inhibitors [1]. Emicizumab is the first non-factor therapy approved for routine prophylaxis in patients with severe or moderate HA, independent of their inhibitor status [8]. Functioning as a recombinant, humanized, bispecific monoclonal antibody, it bridges activated factor IX (FIXa) and factor X (FX), thereby reproducing the cofactor function of activated FVIII (FVIIIa) and restoring hemostatic activity [9]. Unlike FVIII concentrates, emicizumab has no sequence homology with FVIII, allowing it to remain fully effective in patients who have developed FVIII inhibitors [10].

Evidence from the comprehensive HAVEN trial program has consistently demonstrated that emicizumab provides robust protection against bleeding in both inhibitor and non-inhibitor populations, with efficacy comparable to or exceeding that of traditional factor prophylaxis [9, 11–14]. Emicizumab’s clinical impact extends beyond its mechanism of action and effectiveness. Its subcutaneous administration and flexible maintenance dosing schedules, weekly, every 2 weeks, or every 4 weeks, offer a substantial reduction in treatment burden [15]. These advantages are supported by a favorable pharmacokinetic profile featuring a long half-life of approximately 30 days [16].

Real-world prospective data remain essential to understanding how emicizumab performs across diverse clinical settings, particularly in pediatric populations and regions where evidence remains limited. This prospective study aims to evaluate the clinical outcomes, joint health, and functional status of children with HA receiving emicizumab prophylaxis over a 12-month period, providing valuable insight into the therapy’s effectiveness in routine care.

## Materials and methods

### Study design and setting

This prospective cohort study was conducted at the outpatient Hematology Clinic, Zagazig University, in collaboration with the Pediatric Department, Kafrelsheikh University Hospital, and Mansoura Health Insurance Hospital between July 2024 and January 2026. This study was conducted in accordance with the principles of the Declaration of Helsinki. Ethical approval was obtained from the Scientific Research Ethical Committee, Faculty of Medicine, Kafrelsheikh University (Approval No. MKSU 50–6–7). Written informed consent was obtained from parents or legal guardians before enrollment.

The study included 72 children younger than 18 years, consisting of 70 patients with severe HA (FVIII:C < 1 IU/dL) and 2 patients with moderate hemophilia A (FVIII:C 1–5 IU/dL) who presented with a severe clinical phenotype characterized by recurrent spontaneous bleeding and target-joint involvement. Eligible patients included those with current FVIII inhibitors (> 0.6 BU by Bethesda assay) and those without inhibitors who had clinical indications for subcutaneous prophylaxis, including difficult venous access, a history of internal or intracranial hemorrhage, according to our national protocol. Children older than 18 years and patients with other types of hemophilia were excluded. All patients received subcutaneous emicizumab with a loading dose of 3 mg/kg once weekly for 4 weeks, followed by 3 mg/kg every 2 weeks as maintenance doses. Target joints were defined, according to the International Society on Thrombosis and Haemostasis (ISTH), as joints with three or more spontaneous bleeds within a consecutive 6-month period. Target-joint resolution was defined as two or fewer bleeds into the same joint within a consecutive 12-month period [17].

### Clinical assessments and outcomes

At baseline and after 12 months of emicizumab prophylaxis, each participant underwent a comprehensive clinical evaluation that included a detailed medical history and a focused physical examination. Bleeding control was assessed using the annualized bleeding rate (ABR), which served as the primary clinical indicator of prophylactic effectiveness. Joint status was evaluated with the Hemophilia Joint Health Score version 2.1 (HJHS 2.1), in which the six index joints, both ankles, knees, and elbows, were examined for swelling, pain, crepitus, muscle atrophy, strength, and limitations in flexion or extension, in addition to an assessment of gait [18]. The HJHS 2.1 generates a total score ranging from 0, reflecting optimal joint health, to 124, indicating severe impairment. HJHS 2.1 assessments were performed by trained and experienced specialized physicians of the Rehabilitation Physiotherapy Department at the participating centers. The same clinician assessed the same patient at baseline and follow-up to reduce inter-observer variability.

Functional capability was further examined using the Functional Independence Score in Hemophilia (FISH), which measures performance across eight daily activities spanning self-care, transfers, and locomotion [19, 20]. Each task is rated from 1 to 4 based on the level of assistance required, providing a total score between 8 and 32, where higher scores correspond to greater functional independence. Collectively, these clinical assessments provided an integrated view of bleeding outcomes, joint status, and physical functioning during the first year of emicizumab prophylaxis.

### Statistical analysis

Data were analyzed using SPSS version 26. Continuous variables were summarized as mean ± SD or median with interquartile range (IQR), as appropriate. Within-group comparisons over time were performed using paired *t*-tests for normally distributed variables and Wilcoxon signed-rank tests for non-normally distributed variables. Between-group comparisons (inhibitor vs non-inhibitor) used the Mann–Whitney *U* test for continuous variables and chi-square tests for categorical variables. Correlations were evaluated using Pearson’s correlation coefficient. A two-sided *P* value < 0.05 was considered significant.

## Results

### Baseline characteristics

Seventy-two male children with HA were included, with a mean age of 6.7 ± 4.7 years (range, 1 to 17). Thirty-seven children (51.39%) had current FVIII inhibitors. Target joints were present in 50 children (69.44%). All patients had been receiving on-demand therapy before transition to emicizumab prophylaxis. Two children (2.78%) were classified as moderate with a severe bleeding phenotype (Table 1).

**Table 1:** Baseline characteristics of the study population (*N* = 72)

Characteristic	Value
Age, years—mean ± SD (range)	6.7 ± 4.7 (1–17)
Age category—no. (%)	
< 4 years	29 (40.2)
≥ 4 years	43 (59.8)
Weight, kg—mean ± SD (range)	26.85 ± 17.91 (10–70)
Sex—no. (%)	72 (100) male
Annualized bleeding rate	
Median (IQR)	40 (36–48)
Mean (SD)	40.14 (11.24)
Target joints—no. (%)	
Present	50 (69.44)
Absent	22 (30.56)
Factor VIII severity—no. (%)	
Severe (FVIII:C < 1 IU/dL)	70 (97.22)
Moderate (FVIII:C 1–5 IU/dL)	2 (2.78)
FVIII inhibitors—no. (%)	
Positive	37 (51.39)
Negative	35 (48.61)

*SD* denotes standard deviation; *kg*, kilograms

### Bleeding outcomes

After 12 months of emicizumab prophylaxis, the median ABR decreased from 40 (IQR, 36 to 48) to 0 (IQR, 0 to 0) (*P* < 0.001). A total of 57 children (79.16%) experienced zero treated bleeds during follow-up, while 62 (86.11%) had zero treated joint bleeds. Among children with target joints at baseline, 45 (90%) experienced zero treated target-joint bleeds during prophylaxis, and target-joint resolution was observed in all patients after one year. Seventeen bleeding episodes were recorded in 15 patients, where 14 were traumatic, 2 occurred after tooth extraction, and 1 was spontaneous hematuria (Table 2).

**Table 2:** Bleeding outcomes after 12 months of emicizumab prophylaxis

Outcome	After emicizumab (12 months)
Patients with ≥ 1 bleeds—no. (%)	15 (20.88)
Patients with 1 bleed—no. (%)	12 (16.70)
Patients with 2 bleeds—no. (%)	3 (4.18)
Patients with zero bleeds—no. (%)	57 (79.16)
Annualized joint bleeding rate (AJBR)	
Median (IQR)	0.00 (0.00–0.00)
Mean (SD)	0.25 (0.52)
Annualized joint bleeding rate (AJBR)	
Median (IQR)	0 (0–0)
Patients with zero treated joint bleeds—no. (%)	62 (86.11)
Patients with ≥ 1 treated joint bleed—no. (%)	10 (13.89)
Treated target-joint bleeding	
Median (IQR)^a^	0 (0–0)
Patients with zero treated target-joint bleeds—no. (% of baseline target-joint subgroup)	45 (90.0)
Patients with ≥ 1 treated target-joint bleed—no. (% of baseline target-joint subgroup)	5 (10.0)
Cause of bleeding (*N* = 17)	
Traumatic	14 (82.35)
Tooth extraction	2 (11.76)
Spontaneous (hematuria)	1 (5.88)
Site of bleeding (*N* = 17)	
Knee	6 (35.29)
Ankle	2 (11.76)
Big toe/finger	2 (11.76)
Tooth extraction-related	2 (11.76)
Hematuria	1 (5.88)
Epistaxis after trauma	1 (5.88)
Chin after trauma	1 (5.88)
Mucosal bleeding	1 (5.88)
Ear bleeding after trauma	1 (5.88)

^a^Target-joint analyses are based on children with target joints at baseline (*n* = 50)

### Joint health

**Fig. 1:**
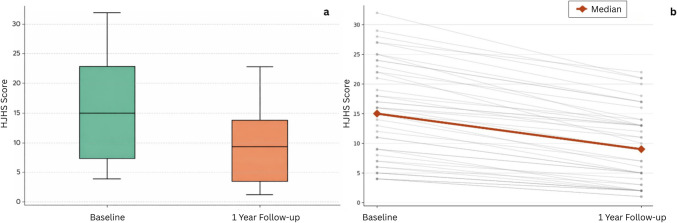
Changes in Hemophilia Joint Health Score (HJHS 2.1) after 12 months of emicizumab prophylaxis. **a** Distribution of HJHS scores at baseline and after 12 months. **b** Individual patient trajectories and median change in HJHS score from baseline to 12 months

HJHS improved after prophylaxis. The median score decreased from 15 (IQR, 7–22) at baseline to 9 (IQR, 3–14) at 12 months (*P* < 0.001). Improvements were observed in both age groups (< 10 years and ≥ 10 years) (Table 3 and Fig. 1).

**Table 3:** HJHS 2.1 at baseline and after 12 months

**Group**	***N***^a^	**Baseline—median (IQR)**	**Range**	**After 12 months—median (IQR)**	**Range**	***P* value**
All patients	43	15 (7–22)	4–32	9 (3–14)	1–22	< 0.001
Age < 10 years	22	7.5 (5–12)	4–21	3 (2–5.25)	1–13	< 0.001
Age ≥ 10 years	21	22 (16.5–26)	12–32	14 (11–17.5)	5–22	< 0.001

^a^HJHS analyses included patients with available paired assessments

#### Functional outcomes

At baseline, the mean total FISH score was 20.65 ± 4.25 out of 32, where the lowest baseline domain scores were recorded in squatting (2.02 ± 0.94), running (2.07 ± 0.91), and stair climbing (2.44 ± 0.73). The highest baseline domain score was eating and grooming (3.21 ± 0.74). After 12 months of emicizumab prophylaxis, functional performance showed marked improvement, with the mean total FISH score rising to 26.02 ± 3.57, reflecting an overall gain of 5.37 points compared with baseline (Supplementary Table 1). All eight assessed activities demonstrated statistically significant enhancement (*P* < 0.001 for all). The greatest improvements were observed in higher-demand tasks, including squatting, running, and stair climbing. Domains with relatively preserved baseline scores also showed measurable improvement, including eating and grooming (*Δ* =  + 0.53) and dressing (*Δ* =  + 0.56) (*P* < 0.001) (Table 4).

**Table 4:** FISH total and domain scores at baseline and after 12 months

Domain	Baseline (mean ± SD)	After 12 months (mean ± SD)	*P* value
Eating and grooming	3.21 ± 0.74	3.74 ± 0.49	< 0.001
Bathing	2.81 ± 0.66	3.60 ± 0.54	< 0.001
Dressing	2.86 ± 0.71	3.42 ± 0.63	< 0.001
Chair transfer	2.51 ± 0.77	3.21 ± 0.64	< 0.001
Squatting	2.02 ± 0.94	2.79 ± 0.77	< 0.001
Walking	2.72 ± 0.70	3.44 ± 0.55	< 0.001
Stair climbing	2.44 ± 0.73	3.24 ± 0.64	< 0.001
Running	2.07 ± 0.91	2.84 ± 0.79	< 0.001
Total FISH score	20.65 ± 4.25	26.02 ± 3.57	< 0.001

*SD*, standard deviation

#### Outcomes by inhibitor status

The analysis of outcomes stratified by FVIII inhibitor status reveals that inhibitor-positive and -negative children achieved nearly identical clinical benefits from emicizumab prophylaxis. At baseline, both groups had a median ABR of 40 with no statistically significant difference (*Z* =  − 0.626; *P* = 0.531). After 1 year of emicizumab prophylaxis, the median ABR for both groups decreased to 0. Within-group changes from baseline to 12 months were highly significant for both subgroups (Wilcoxon *P* < 0.001 for each). Bleeding phenotype after prophylaxis also showed comparable outcomes. Zero treated bleeds were comparable between non-inhibitor and inhibitor-positive children, with no significant difference (*P* = 0.321). Similarly, there was no difference between zero treated joint bleeds in non-inhibitor and inhibitor-positive children (*P* = 0.437). Among children with baseline target joints (*n* = 50), zero target-joint bleeds were achieved in 21 of 23 non-inhibitor children (91.30%) and 24 of 27 inhibitor-positive children (88.89%), with no significant difference (*P* = 0.777) (Table 5).

**Table 5:** Association of FVIII inhibitor status with outcomes

Outcome	Non-inhibitor (*n* = 35)	Inhibitor (*n* = 37)	Test statistic	*P* value
ABR—median (IQR)				
Baseline	40 (30–48)	40 (36–48)	*Z* = − 0.626	0.531
After 12 months	0 (0–1)	0 (0–0)	*Z* = − 1.005	0.315
Within-group *P* value (Wilcoxon)	< 0.001	< 0.001	—	—
HJHS—median (IQR)				
Baseline	15 (5–21.25)	16 (9–23.75)	*Z* = − 0.826	0.409
After 12 months	8.5 (2–13.75)	9.5 (4.5–14)	*Z* = − 0.409	0.683
Within-group *P* value (Wilcoxon)	< 0.001	< 0.001	—	—
AJBR after prophylaxis—median (IQR)	0 (0–0)	0 (0–0)	*Z* = − 0.771	0.441
Treated target-joint bleeding after prophylaxis—median (IQR)	0 (0–0)	0 (0–0)	*Z* = − 0.281	0.779
FISH—mean ± SD				
Baseline	20.83 ± 3.63	19.23 ± 3.09	*t* = 1.715	0.094
After 12 months	27.06 ± 3.75	25.28 ± 3.31	*t* = 1.608	0.117
Within-group *P* value (paired *t*-test)	< 0.001	< 0.001	—	—
Treated bleeding episodes after 12 months				
Zero bleeding episodes—no. (%)	26 (74.29%)	31 (83.78%)	—	0.321
≥ 1 bleeding episodes—no. (%)	9 (25.71%)	6 (16.22%)	—	
Treated joint bleeding after 12 months				
Zero bleeding episodes—no. (%)	29 (82.86)	33 (89.19)	—	0.437
≥ 1 bleeding episodes—no. (%)	6 (17.14)	4 (10.81)	—	
Treated target-joint bleeding after 12 months				
Zero treated target-joint bleeds—no. (%)^a^	21 (91.30)	24 (88.89)	—	0.777
≥ 1 treated target-joint bleed—no. (%)^a^	2 (8.70)	3 (11.11)	—	

*ABR*, annualized bleeding rate; *AJBR*, annualized joint bleeding rate; *HJHS*, Hemophilia Joint Health Score; *FISH*, Functional Independence Score in Hemophilia; *IQR*, interquartile range; *SD*, standard deviation

^a^Percentages are calculated within baseline target-joint subgroups

Joint health outcomes followed the same pattern. Among patients who had paired HJHS assessments, both non-inhibitor and inhibitor-positive patients showed a decrease in median HJHS (*P* < 0.001 for all). As with the ABR results, there were no statistically significant differences between groups at baseline or follow-up (*P* > 0.40 for both comparisons). Functional outcomes were also remarkably consistent. The non-inhibitor group improved from a baseline mean FISH score of 20.83 ± 3.63 to 27.06 ± 3.75, while the inhibitor group improved from 19.23 ± 3.09 to 25.28 ± 3.31. Both within-group improvements were statistically significant (paired *t*-test *P* < 0.001), and the between-group differences in improvement magnitude were not statistically significant (baseline *P* = 0.094; follow-up *P* = 0.117) (Table 5).

### Safety outcomes

Regarding safety outcomes, emicizumab was well-tolerated throughout the 12-month follow-up period. A total of five local injection-site reactions were reported, including local pain in two patients and local erythema in three patients. No thromboembolic events, serious adverse events, or treatment discontinuation were reported during the study period.

## Discussion

In this prospective study of 72 children with HA, emicizumab prophylaxis achieved a profound reduction in bleeding, with the median ABR falling from 40 at baseline to 0 after 12 months, and nearly 80% of participants experienced no treated bleeds. Significant improvements were also observed in joint health, with median HJHS scores decreasing from 15 to 9 and complete resolution of all target joints, and in functional independence, with the mean FISH score rising from 20.65 to 26.02. Notably, children with and without FVIII inhibitors achieved comparable outcomes across all bleeding, joint, and functional measures, underscoring the consistent efficacy of emicizumab irrespective of inhibitor status. These outcomes underscore the profound clinical impact of this therapy and validate its central place in contemporary hemophilia care [5]. The dramatic reduction in bleeding frequency, improvement in joint health, and enhanced functional independence reflected the pharmacological effectiveness of emicizumab prophylaxis [8, 21].

The significant reduction in ABR after 1 year of emicizumab demonstrates the effectiveness of prophylaxis in protection against bleeding, which is consistent with the highest-quality clinical evidence. The HAVEN 2 trial focused specifically on children with FVIII inhibitors and showed that treated ABR was reduced to approximately 0.3, and 77% of participants experienced no treated bleeds. Our study showed zero treated bleeds in 79.16% of children, aligning with these outcomes in the HAVEN 1–5 trials [9, 11–14].

Real-world pediatric studies have similar findings, where children receiving emicizumab achieve zero treated bleeding events, and ABRs generally fall below 1.0 in treated populations. The concordance between trial data and routine clinical results suggests that emicizumab maintains its efficacy across diverse healthcare settings, including resource-limited environments. Its subcutaneous administration and fixed dosing schedule contribute to this consistency by reducing adherence challenges inherent to intravenous prophylaxis [5]. Our results are aligned with current guidelines that recommend sustained prophylaxis to prevent hemarthroses, maintain joint health, and safeguard physical development [21].

The significant improvements observed in joint and functional health reflect the clinical significance of persistent bleed control. The reduction in HJHS and the complete resolution of target joints in all affected children indicate a strong protective effect on the musculoskeletal system. Similar trends have been documented in the long-term follow-up of the HAVEN trials, where more than 99% of target joints demonstrated sustained resolution [12]. The near-control of joint bleeding in this cohort provides compelling evidence that early treatment with emicizumab may alter the progression of arthropathy in children with HA [22, 23].

The parallel increase in functional activity, as reflected in the improvement in mean FISH scores, further supports that bleed prevention translates into clear clinical benefit. Children receiving emicizumab prophylaxis notably gained in physically demanding domains, including squatting, running, and stair climbing, activities that are directly affected by joint integrity and pain. These improvements suggest that emicizumab’s impact extends to enabling more effective physical participation, potentially enhancing psychosocial development, confidence, and overall quality of life [24]. Similar patterns have been reported in structured quality-of-life assessments from the HAVEN program, supporting the link between bleed control, functional gains, and improved day-to-day well-being [9, 11–14].

One of the most notable elements of this study is the equivalent efficacy observed in both inhibitor-positive and inhibitor-negative children. This result is entirely consistent with the molecular design and pharmacology of emicizumab [25]. As a humanized bispecific monoclonal antibody that binds FIXa and FX to reproduce the cofactor function of FVIIIa, emicizumab bypasses the antigenic sites targeted by FVIII alloantibodies [9, 10]. Its clinical activity is therefore unaffected by inhibitor titers, enabling uniform prophylactic coverage across patient groups that historically would have had drastically different treatment outcomes. The consistent findings across inhibitor statuses reinforce the conceptual shift introduced by emicizumab, that inhibitors no longer dictate the severity of clinical phenotype or the feasibility of achieving reliable prophylaxis [26, 27]. The drug’s mechanism results in steady-state plasma concentrations that produce an FVIII-equivalent activity generally estimated at 10–20% [11, 28]. Clinically, emicizumab effectively converts severe HA phenotypes into milder phenotypes through dramatically reducing spontaneous bleeding episodes in both groups [26, 27]. The parallel improvements in ABR, HJHS, and FISH across inhibitor categories in this cohort further confirm that bypassing the deficient factor pathway successfully mitigates the historical challenges associated with inhibitor development [5].

### Study strengths and limitations

This study offers several important strengths. First, it provides prospective real-world data from a pediatric population with a substantial proportion of inhibitor-positive patients, a group that historically has had limited treatment options and poor outcomes with bypassing agent prophylaxis [12]. Second, the evaluation of multidimensional clinical outcomes, including joint health and functional independence, presents a comprehensive assessment of therapeutic benefit beyond bleeding metrics. Finally, the representation of a low- to middle-income healthcare setting highlights the feasibility of integrating emicizumab into diverse clinical infrastructures, where venous access challenges and treatment burden frequently hinder optimal prophylaxis [29, 30].

The study also has limitations. Its 12-month follow-up period, although adequate for assessing bleed control, is too short to determine the long-term impact on joint health or the durability of functional gains. Longer-term observation is necessary to evaluate whether early bleed suppression continues to protect joints through adolescence and adulthood. An additional limitation was that HJHS assessors were not blinded to treatment status in this prospective real-world study. This could impose a degree of observer bias that cannot be completely excluded.

### Clinical implications

The results strengthen the case for early emicizumab prophylaxis in children with severe HA. The ease of subcutaneous administration eliminates the need for central venous access devices and simplifies adherence, which is especially valuable in young children. For inhibitor-positive patients, the ability to achieve bleed control equivalent to that of non-inhibitor patients represents a significant advance and redefines the expectations for long-term outcomes. For inhibitor-negative patients, emicizumab offers a highly effective and less burdensome alternative to frequent intravenous FVIII infusions.

### Future directions

Future research should prioritize long-term follow-up to determine the durability of joint protection and functional improvements in children who initiate emicizumab early in life. Ongoing efforts to personalize emicizumab dosing using pharmacokinetic modeling tools may further enhance efficacy and reduce drug wastage. Finally, emerging therapies such as next-generation FVIII mimetics and rebalanced hemostasis agents will need to be compared with emicizumab to determine their relative benefits and roles in future care algorithms [31, 32].

## Conclusion

In this prospective pediatric cohort, emicizumab prophylaxis resulted in near-complete control of bleeding, marked improvement in joint health, and substantial gains in functional independence over one year of follow-up. The consistency of benefit across inhibitor-positive and inhibitor-negative children underscores the robustness of emicizumab’s mechanism and its ability to overcome one of the most significant historical challenges in hemophilia care. The resolution of all target joints and the observed functional improvements suggest that early and sustained emicizumab prophylaxis may favorably alter the long-term musculoskeletal trajectory of children with severe HA. These findings reinforce international guideline recommendations advocating universal prophylaxis and support the integration of emicizumab as a cornerstone of pediatric hemophilia management, particularly in settings where venous access and adherence remain major barriers to optimal care.

## Supplementary Information

Below is the link to the electronic supplementary material.

ESM 1 DOCX (16.4 KB)

## Data Availability

Data supporting our findings are available upon reasonable request from the corresponding author.

## Abbreviations

ABR: Annualized bleeding rate
AJBR: Annualized joint bleeding rate
BU: Bethesda unit(s)
FIXa: Activated factor IX
FISH: Functional Independence Score in Hemophilia
FVIII: Factor VIII
FVIII:C: Factor VIII activity (coagulant activity)
FX: Factor X
FVIIIa: Activated factor VIII
HA: Hemophilia A
HJHS: Hemophilia Joint Health Score
HJHS 2.1: Hemophilia Joint Health Score, version 2.1
IQR: Interquartile range
SD: Standard deviation
WFH: World Federation of Hemophilia

## References

[1] Srivastava A, Brewer AK, Mauser-Bunschoten EP, et al (2013) Guidelines for the management of hemophilia. Haemophilia 19:. 10.1111/J.1365-2516.2012.02909.X 10.1111/j.1365-2516.2012.02909.x 22776238

[2] Graham A, Jaworski K (2014) Pharmacokinetic analysis of anti-hemophilic factor in the obese patient. Haemophilia 20:226–229. 10.1111/HAE.12300 24252161 10.1111/hae.12300

[3] Peyvandi F, Mannucci PM, Garagiola I et al (2016) A randomized trial of factor VIII and neutralizing antibodies in hemophilia A. N Engl J Med 374:2054–2064. 10.1056/NEJMOA1516437 27223147 10.1056/NEJMoa1516437

[4] Fischer K, Lewandowski D, Marijke van den Berg H, Janssen MP (2012) Validity of assessing inhibitor development in haemophilia PUPs using registry data: the EUHASS project. Haemophilia 18:. 10.1111/J.1365-2516.2011.02687.X 10.1111/j.1365-2516.2011.02687.x 22044445

[5] Alcedo Andrade PE, Manucci PM, Kessler CM (2024) Emicizumab: the hemophilia A game-changer. Haematologica 109:1334–1347. 10.3324/HAEMATOL.2022.282099 37916312 10.3324/haematol.2022.282099 PMC11063855

[6] Walsh CE, Soucie JM, Miller CH (2015) Impact of inhibitors on hemophilia A mortality in the United States. Am J Hematol 90:400–405. 10.1002/AJH.23957 25616111 10.1002/ajh.23957

[7] D’Angiolella LS, Cortesi PA, Rocino A et al (2018) The socioeconomic burden of patients affected by hemophilia with inhibitors. Eur J Haematol 101:435–456. 10.1111/EJH.13108 29889317 10.1111/ejh.13108

[8] Manco-Johnson MJ, Abshire TC, Shapiro AD et al (2007) Prophylaxis versus episodic treatment to prevent joint disease in boys with severe hemophilia. N Engl J Med 357:535–544. 10.1056/NEJMOA067659 17687129 10.1056/NEJMoa067659

[9] Oldenburg J, Mahlangu JN, Kim B et al (2017) Emicizumab prophylaxis in hemophilia A with inhibitors. N Engl J Med 377:809–818. 10.1056/NEJMOA1703068 28691557 10.1056/NEJMoa1703068

[10] Lenting PJ, Denis VC, Christophe OD (2017) Emicizumab, a bispecific antibody recognizing coagulation factors IX and X: how does it actually compare to factor VIII? Blood 130:2463–2468. 10.1182/BLOOD-2017-08-801662 29042366 10.1182/blood-2017-08-801662

[11] Pipe SW, Shima M, Lehle M et al (2019) Efficacy, safety, and pharmacokinetics of emicizumab prophylaxis given every 4 weeks in people with haemophilia A (HAVEN 4): a multicentre, open-label, non-randomised phase 3 study. Lancet Haematol 6:e295–e305. 10.1016/S2352-3026(19)30054-7 31003963 10.1016/S2352-3026(19)30054-7

[12] Mahlangu J, Oldenburg J, Paz-Priel I et al (2018) Emicizumab prophylaxis in patients who have hemophilia A without inhibitors. N Engl J Med 379:811–822. 10.1056/NEJMOA1803550 30157389 10.1056/NEJMoa1803550

[13] Yang R, Wang S, Wang X et al (2022) Prophylactic emicizumab for hemophilia A in the Asia-Pacific region: a randomized study (HAVEN 5). Res Pract Thromb Haemost. 10.1002/rth2.12670 35284778 10.1002/rth2.12670 PMC8902287

[14] Young G, Liesner R, Chang T et al (2019) A multicenter, open-label phase 3 study of emicizumab prophylaxis in children with hemophilia A with inhibitors. Blood 134:2127–2138. 10.1182/BLOOD.2019001869 31697801 10.1182/blood.2019001869 PMC6908828

[15] Yoneyama K, Schmitt C, Kotani N et al (2018) A pharmacometric approach to substitute for a conventional dose-finding study in rare diseases: example of phase III dose selection for emicizumab in hemophilia A. Clin Pharmacokinet 57:1123–1134. 10.1007/S40262-017-0616-3 29214439 10.1007/s40262-017-0616-3 PMC6061395

[16] Li A, Goodfriend C, Sokol J, Kruse-Jarres R (2019) Patterns and predictors of emicizumab adherence in people with hemophilia. Blood 134:2178–2178. 10.1182/BLOOD-2019-128083

[17] Blanchette VS, Key NS, Ljung LR et al (2014) Definitions in hemophilia: communication from the SSC of the ISTH. J Thromb Haemost 12:1935–1939. 10.1111/jth.12672 25059285 10.1111/jth.12672

[18] Feldman BM, Funk SM, Bergstrom BM et al (2011) Validation of a new pediatric joint scoring system from the International Hemophilia Prophylaxis Study Group: validity of the hemophilia joint health score. Arthritis Care Res (Hoboken) 63:223–230. 10.1002/ACR.20353 20862683 10.1002/acr.20353

[19] Poonnoose PM, Thomas R, Keshava SN et al (2007) Psychometric analysis of the Functional Independence Score in Haemophilia (FISH). Haemophilia 13:620–626. 10.1111/J.1365-2516.2007.01508.X 17880453 10.1111/j.1365-2516.2007.01508.x

[20] Poonnoose PM, Manigandan C, Thomas R et al (2005) Functional Independence Score in Haemophilia: a new performance-based instrument to measure disability. Haemophilia 11:598–602. 10.1111/J.1365-2516.2005.01142.X 16236109 10.1111/j.1365-2516.2005.01142.x

[21] Srivastava A, Santagostino E, Dougall A et al (2020) WFH guidelines for the management of hemophilia, 3rd edition. Haemophilia 26(6):1–158. 10.1111/HAE.14046 32744769 10.1111/hae.14046

[22] Jansen NWD, Roosendaal G, Lafeber FPJG (2008) Understanding haemophilic arthropathy: an exploration of current open issues. Br J Haematol 143:632–640. 10.1111/J.1365-2141.2008.07386.X 18950457 10.1111/j.1365-2141.2008.07386.x

[23] Seuser A, Khayat CD, Negrier C et al (2018) Evaluation of early musculoskeletal disease in patients with haemophilia: results from an expert consensus. Blood Coagul Fibrinolysis 29:509–520. 10.1097/MBC.0000000000000767 30020119 10.1097/MBC.0000000000000767 PMC6125749

[24] Shima M, Nogami K, Nagami S et al (2019) A multicentre, open-label study of emicizumab given every 2 or 4 weeks in children with severe haemophilia A without inhibitors. Haemophilia 25:979–987. 10.1111/HAE.13848 31515851 10.1111/hae.13848 PMC6900083

[25] Jimenez-Yuste V, Shima M, Fukutake K et al (2017) Emicizumab subcutaneous dosing every 4 weeks for the management of hemophilia A: preliminary data from the pharmacokinetic run-in cohort of a multicenter, open-label, phase 3 study (HAVEN 4). Blood 130:86–86. 10.1182/BLOOD.V130.SUPPL_1.86.8628490571

[26] Liu G, Huang K, Li G et al (2022) Real-world experience of emicizumab prophylaxis in young children with hemophilia A: retrospective data from China. Front Pediatr. 10.3389/FPED.2022.992267 36340724 10.3389/fped.2022.992267 PMC9627604

[27] Glonnegger H, Andresen F, Kapp F et al (2022) Emicizumab in children: bleeding episodes and outcome before and after transition to emicizumab. BMC Pediatr. 10.1186/S12887-022-03546-1 35965332 10.1186/s12887-022-03546-1 PMC9377120

[28] Donners AAMT, Rademaker CMA, Bevers LAH et al (2021) Pharmacokinetics and associated efficacy of emicizumab in humans: a systematic review. Clin Pharmacokinet 60:1395–1406. 10.1007/S40262-021-01042-W 34389928 10.1007/s40262-021-01042-w PMC8585815

[29] Kengkla K, Wilairat P, Natesirinilkul R et al (2024) Evaluating the benefits of emicizumab prophylaxis for haemophilia A with inhibitors: a cost-effectiveness and budget impact analysis in Thailand’s upper-middle income setting. Haemophilia 30:1288–1297. 10.1111/HAE.15105 39368064 10.1111/hae.15105

[30] Mannucci PM, Hermans C (2024) Low-dose emicizumab for more equitable access to prophylaxis in resource limited countries. Haemophilia 30:575–576. 10.1111/HAE.14968 38415381 10.1111/hae.14968

[31] Bowyer AE, Hickey K, Kitchen S, Ezban M (2023) A next generation FVIII mimetic bispecific antibody, Mim8, the impact on non-factor VIII related haemostasis assays. Haemophilia 29:1633–1637. 10.1111/HAE.14884 37824563 10.1111/hae.14884

[32] Yamaguchi K, Soeda T, Sato M et al (2020) Pharmacology and pharmacokinetics of NXT007; emicizumab-based engineered Fixa/Fx bispecific antibody with improved properties. Blood 136:19–19. 10.1182/BLOOD-2020-138958

