# Supplementary material for: Emicizumab prophylaxis beyond clinical trials: a multicenter, prospective real-world study of pediatric hemophilia patients with and without inhibitors
Source: Eur J Pediatr. 2026 Apr 11;185(5):255. doi: 10.1007/s00431-026-06872-z (PMC13070065; doi:10.1007/s00431-026-06872-z)
Supplement: Supplementary file 1 — DOCX (16.4 KB) [file 431_2026_6872_MOESM1_ESM.docx]

**Supplementary Table 1.** FISH score proportions at baseline and after a year of receiving prophylaxis

|  | **At baseline** | | | | **After year** | | | |
| --- | --- | --- | --- | --- | --- | --- | --- | --- |
| **Degree of function** | **1** | **2** | **3** | **4** | **1** | **2** | **3** | **4** |
| **Eating and grooming** | 0 | 8 (18.6%) | 18 (41.86%) | 17  (39.53%) | 0 | 1 (2.33%) | 9 (20.93%) | 33 (76.74%) |
| **Bathing** | 0 | 14 (32.56%) | 23 (53.49%) | 6 (13.95%) | 0 | 1 (2.33%) | 15 (34.88%) | 27 (62.79%) |
| **Dressing** | 0 | 14 (32.56%) | 21  (48.84%) | 8 (18.6%) | 0 | 3 (6.98%) | 19  (44.19%) | 21 (48.84%) |
| **Chair** | 2 (4.65%) | 22 (51.16%) | 14 (32.56%) | 5 (11.63%) | 0 | 5 (11.63%) | 24 (55.81%) | 14 (32.56%) |
| **Squatting** | 14 (32.56%) | 18 (41.86%) | 7 (16.28%) | 4 (9.3%) | 1 (2.33%) | 15 (34.88%) | 19 (44.19%) | 8 (18.6%) |
| **Walking** | 2 (4.65%) | 12 (27.91%) | 25 (58.14%) | 4 (9.3%) | 0 | 1 (2.33%) | 22 (51.16%) | 20 (46.51%) |
| **Stair climb** | 2 (4.65%) | 24 (55.81%) | 13 (30.23%) | 4 (9.3%) | 1 (2.33%) | 9 (20.93%) | 23 (53.49%) | 10 (23.26%) |
| **Running** | 13 (30.23%) | 17 (39.53%) | 10 (23.26%) | 3 (6.98%) | 1 (2.33%) | 14 (32.56%) | 19 (44.19%) | 9 (20.93%) |
